# Adjusting for cross-cultural differences in computer-adaptive tests of quality of life

**DOI:** 10.1007/s11136-017-1738-7

**Published:** 2017-12-04

**Authors:** C. J. Gibbons, S. M. Skevington, WHOQOL Group

**Affiliations:** 1 THIS Institute (The Healthcare Improvement Studies Institute), School of Clinical Medicine, University of Cambridge, Cambridge, UK; 2 University of Cambridge Psychometrics Centre, Cambridge, UK; 3 Manchester Centre for Health Psychology and International Hub for Quality of Life Research, Division of Psychological Sciences and Mental Health, University of Manchester, Manchester, M13 PL9 UK

**Keywords:** Quality of life, Computer-adaptive testing, WHOQOL, Cross-cultural, Assessment

## Abstract

**Purpose:**

Previous studies using the WHOQOL measures have demonstrated that the relationship between individual items and the underlying quality of life (QoL) construct may differ between cultures. If unaccounted for, these differing relationships can lead to measurement bias which, in turn, can undermine the reliability of results.

**Methods:**

We used item response theory (IRT) to assess differential item functioning (DIF) in WHOQOL data from diverse language versions collected in UK, Zimbabwe, Russia, and India (total *N* = 1332). Data were fitted to the partial credit ‘Rasch’ model. We used four item banks previously derived from the WHOQOL-100 measure, which provided excellent measurement for physical, psychological, social, and environmental quality of life domains (40 items overall). Cross-cultural differential item functioning was assessed using analysis of variance for item residuals and post hoc Tukey tests. Simulated computer-adaptive tests (CATs) were conducted to assess the efficiency and precision of the four items banks.

**Results:**

Splitting item parameters by DIF results in four linked item banks without DIF or other breaches of IRT model assumptions. Simulated CATs were more precise and efficient than longer paper-based alternatives.

**Discussion:**

Assessing differential item functioning using item response theory can identify measurement invariance between cultures which, if uncontrolled, may undermine accurate comparisons in computer-adaptive testing assessments of QoL. We demonstrate how compensating for DIF using item anchoring allowed data from all four countries to be compared on a common metric, thus facilitating assessments which were both sensitive to cultural nuance and comparable between countries.

**Electronic supplementary material:**

The online version of this article (10.1007/s11136-017-1738-7) contains supplementary material, which is available to authorized users.

## Introduction

Quality of life differs between individuals and across different cultures. Traditional methods of comparing quality of life (QoL) between cultures, for example comparing ordinal summary scores from scales in different cultures, do not allow for nuanced differences in the interpretation of items. Although considerable development work was conducted to ensure that items of the World Health Organisation Quality of Life (WHOQOL) measures were developed and validated in a manner that enhanced semantic and conceptual equivalence [1, 2], previous research has identified issues with metric equivalence between language versions of the WHOQOL-100 questionnaire [3]. The current study aims to evaluate the metric equivalence of a 40-item bank derived from the WHOQOL-100 and to statistically compensate for different response behaviours between cultures.

Item response theory (IRT) describes the probabilistic relationship between items and test takers, such that an item which measures a high level of QoL is more likely to be affirmed by a person who actually has a high level of QoL than someone with a lower level [4]. It is possible that items can vary in their relationship to the underlying trait across different demographic groups, meaning that, as well as measuring the underlying construct, the item measures a nuance in the interpretation of that item between these demographic groups. This phenomenon is known as differential item functioning (DIF) and, if present, causes problems for the interpretation of assessments made between groups [5, 6].

Where DIF is absent between groups, people with the same level of QoL will have the same chance of responding in a certain way to the item. Where DIF is present for an item, then people from different cultures with the same level of QoL will have a different likelihood of responding in a certain way to that item. This difference indicates a nuance in the interpretation of the item. Left unadjusted, DIF interferes with fit to a psychometric model and precludes the item’s use in comparative assessments between countries.

Differential item functioning does not necessarily provide substantive evidence of poor cross-cultural validity. The presence of DIF may be attributed to either poor cross-cultural validity or expected nuances in cultural understanding of QoL. Evidence of cross-cultural validity must therefore be established separately using a rigorous process of cultural adaptation, such as the spoke-wheel methodology utilized by the WHOQOL group [1, 2] or the FACIT translation methodology [7].

Where DIF is indicated for an item that has shown to be cross-culturally valid, then efforts ought to be made to preserve that item. Where DIF is shown to be interfered with accurate comparisons between groups, it is common practice to remove affected items from the item banks or questionnaires [8]. The item removal strategy has been adopted in previous studies which evaluated the cross-cultural measurement properties of WHOQOL items [3]. Although this strategy successfully improves psychometric model fit, it is at risk of narrowing the range of measurement by removing items that, for some groups, are relevant [9].

There are alternatives to an item removal approach, which can retain items and adjust for the differences in item interpretation between groups. Known as ‘item anchoring’ [6], the methodology allows item parameters to vary by demographic group where DIF is present, but retains shared item parameters for items where DIF is absent. Compensating for DIF in this way can allow accurate measurement within and between countries [6]. Similar methods have also been used to link different scales together on a common metric [10, 11].

While the precision and accuracy of cross-cultural measurement can be improved using item anchoring techniques nested within an item response theory framework, assessments can be improved further using computer-adaptive testing (CAT), a special type of questionnaire administration. In contrast to fixed-length questionnaires, it uses computational algorithms to intelligently and interactively match the participant with the most relevant item for them. Using CAT to select a suitable subset of items for assessment can lead to significant gains in assessment efficiency (i.e. the number of items which need to be administered before a certain level of psychometric reliability is reached), and precision, as information is maximized for each individual [8, 12, 13]. Computer-adaptive testing has been described as “the most exciting development in health assessment” [14] and is now supported by open-source software to facilitate the implementation of CATs in practice [15]. The ability for CATs to significantly reduce assessment burden while retaining, or even improving, measurement accuracy is the motivation for focussing on administration using this methodology in the current paper. We provide an example of validated QoL assessments using CAT methodology which can be found at ehealthtools.co.uk.

In the current paper, we demonstrate the use of item anchoring to resolve DIF identified using a single-parameter IRT model (i.e. the partial credit model) using and item bank derived from the WHOQOL-100 [16], for the purposes of CAT. We show that cross-cultural research can be improved with more robust and efficient assessments using culturally valid computer-adaptive tests [17].

## Methods

We compared data from four cultures in countries from different world regions to illustrate our method. Analysis was based on a previous-designed 40-item bank designed for use in the UK [16]. We then randomly selected three other cultures from 14 others available, which had simultaneously developed language versions in accordance with the WHOQOL Group’s common, internationally agreed protocol. All items were culturally adapted and translated by potential users during development in each centre. Diverse cultures from Russia (St Petersburg), Zimbabwe (Harare), and India (Madras) were chosen for the current study using a ‘true’ random number generator (Random.org).

## Measures

### WHOQOL-100

The World Health Organization Quality of Life Assessment (100-item version) (WHOQOL-100) is a generic measure of subjective quality of life comprising 100 items rated on five-point Likert interval scales, which were specially designed for this measure [18]. The 25 topics or facets of QoL are known to be relevant to groups of people with different ages, cultures, genders, health status, and for most major physical and psychological diagnostic groups [19]. Cross-cultural validity in terms of item translation and content has been well established in previous investigations [1, 2].

In this study, we used items from the WHOQOL-100 item bank which was validated for a UK population in a previous study [16]. The item bank consisted of 40 items arranged into a four-domain structure (physical QoL, psychological QoL, social QoL, and environmental QoL) to mirror the structure of the WHOQOL-BREF [16, 20].

## Analysis

### Item response theory

We assess the advanced psychometric criteria, and estimated item bank parameters using the partial credit ‘Rasch’ model (PCM) [21]. Model assumptions were examined for each item and, where necessary, items within the bank were removed or modified. Tests of model assumptions, as well as their solutions, are discussed below, and further information relating to the process of Rasch analysis is described in greater length elsewhere [6, 22, 23].

### Differential item functioning

Differential item functioning (DIF) was assessed for each of the cultures included in the analysis. The presence of DIF was identified using analysis of variance (ANOVA). Two types of DIF can be identified: uniform DIF, where the difference between the groups is constant across all levels of the underlying phenomenon (in this case, QoL); and non-uniform DIF, where the relationship between groups differs along the QoL continuum. Differential item functioning is identified where ANOVA interactions are significant, following Bonferroni correction for multiple comparisons [24, 25]. The number of comparisons was equal to the number of items in each bank. Because the current analysis compared effects between more than two groups, post hoc Tukey tests were conducted to establish between which cultures statistically significant DIF effects were evident. The ANOVA method shows favourable performance compared to Mantel–Haenszel and logistic regression approaches for detecting uniform DIF, though logistic regression showed better performance for detecting non-uniform DIF [26].

### Item fit and fit residual

Individual item fit to the partial credit model is assessed using Chi-square tests between the residuals and the model. A non-significant interaction suggests that the data are consistent with the expectations of the model. Bonferroni corrections are applied to account for multiple comparisons [27]; in each instance, the number of comparisons is equal to the number of items in the bank.

### Category threshold ordering

When IRT is used to analyse scale data that has employed a Likert-type response, a probability value is given to each response at all levels of the underlying construct. For categories to be correctly ordered, there must be a point along the continuum of the underlying construct where it is the most likely response. Violation of this condition results in disordered threshold values that negatively impact model fit and prohibit CAT assessment. Disordered category thresholds can be rectified by employing a new scoring strategy [28]. For example, if categories “3—Agree” and “4—Strongly agree” were disordered, the item categories may be rescored from 0 to 1-2-3-4 to 0-1-2-3-3.

### Local dependency

Item response theory assumes that item responses are conditional solely on the level of underlying construct that a person has (e.g. how high or low their quality of life is). Where this assumption is held, there is said to be local independence of items. Local dependency is assessed using Yen’s Q3 correlation between item residuals. A residual correlation greater than + 0.20 indicates local dependency [29, 30].

### Unidimensionality

Instruments that are calibrated to item response theories must measure only a single underlying construct. Dimensionality of the WHOQOL scales has been assessed by conducting a principal components analysis of the item residuals followed by an independent t test on the first factor of the residuals [31]. The *t* tests are used to compare the estimates for each person and the percentage of the tests outside of the range ± 1.96. If the number of significant *t* tests is lower than 5% of the total sample (or the lower bound of the 95% confidence interval is below 5%), then the scale is considered to be unidimensional [23].

### Item anchoring

The term item anchoring describes the process by which the parameters of items with DIF are allowed to vary, while item calibrations for the other items which displayed DIF remain constant across each country [6]. Common ‘anchored’ items ensure that direct comparisons can be made between cultures, while the parameter values for items with DIF vary to accommodate cultural differences in the metric comparison [32]. Items are anchored at the threshold level.

### Reliability

Reliability is assessed initially using the person separation index which, when data are normally distributed, is analogous to Cronbach’s Alpha [27].

### Model fit

Scale fit to the partial credit model is assessed with Chi-square tests between the model and the scale data [27]. However, test can be problematic for assessing overall scale fit because of a tendency to uncover spuriously significant relationships, especially in larger samples. Model fit will therefore be assessed, but in the event of a significant Chi-square interaction, model fit will be deemed acceptable if all the assumptions described above are met. This is because of the tendency for Chi-square analyses to commit type I errors with larger sample sizes, the large sample size in the current study (*N* = 1332) increases the risk of type I error significantly [17].

### Computerized adaptive testing simulation

To establish the performance of the item banks relative to each other, and to the paper-based version of the WHOQOL-100, we conducted the simulation using the CAT FIRESTAR engine [33]. The first item that the CAT administered for each domain was the item with the greatest information function at the distribution mean. We used the normal IRT scaling constant (1.7) [34]. We conducted 1000 iterations of the CAT using a normal distribution of scores representative of the general population.

Our stopping rule stated that once the test had matched an equal level of reliability from the published WHOQOL paper-based measures (WHOQOL-BREF and WHOQOL-100) [20, 35], the CAT simulation would stop. For example, if the published reliability for the Psychological QoL domain was 0.82, we set the stopping rule standard error to 0.42 (which is roughly equivalent to Cronbach’s Alpha *α* = 0.82, assuming a normal distribution of scores) and the mean number of items administered was compared with the length of the paper-based questionnaire. Simulations were also conducted with stopping rules of standard errors of 0.55 and 0.32 (equivalent to Cronbach’s Alpha (*α*) 0.70 and 0.90, where the standard deviation of the trait is equal to 1).

Firestar uses a Bayesian expected *a posteriori* (EAP) theta estimator (with a prior distribution of *N*(0,1)) and the maximum posterior-weighted information (MPWI) item selection criterion. The MPWI selects items based on the information function weighted by the posterior distribution of construct scores [36]. This criterion has been shown to provide excellent measurement information for CAT using polytomous items (e.g. those scored on a Likert-type response scale) [36]. Simulations were conducted using simulated respondents at discrete intervals (0.10) along the theta continuum (from − 4 to 4).

Item response theory analyses were conducted using the Rasch unidimensional measurement models 2030 (RUMM2030) software [37]. Computerized adaptive testing simulation was conducted using an adapted FIRESTAR code generator for the R Statistical Computing language [33, 38].

## Results

### Differential item functioning

**Fig. 1:**
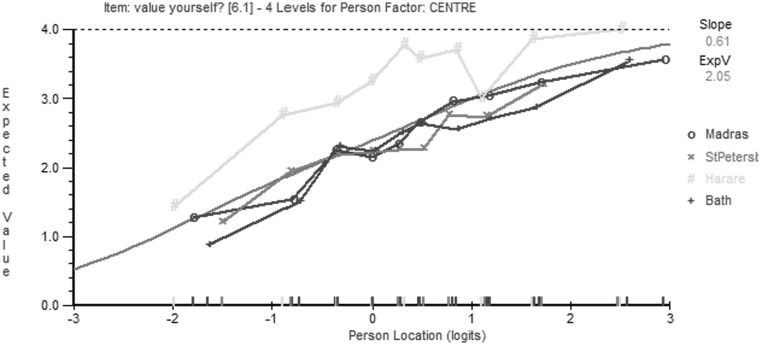
Example of DIF between countries for item f6.1 “How much do you value yourself?” This figure demonstrated clear DIF for item F6.1 “How much do you value yourself?” between Zimbabwe and all other countries. This indicates that at all person locations (levels of psychological quality of life) people from Zimbabwe score more highly on this item than people from the UK, Russia, and India

Across the four item banks containing 40 items, a total of 30 items (75% of the item bank) demonstrated DIF between at least two cultures. An overall summary of DIF occurrences between countries and domains is provided in Table 1. Information on which item displayed DIF and the groups affected is shown in Table 2. A graphical example of DIF is shown in Fig. 1 for the item f6.1 “How much do you value yourself?” In this item, DIF is present between Zimbabwe and all three other countries.

**Table 1:** Summary of DIF occurrences between countries and domains

	Physical	Psychological	Social	Environmental
UK				
Total items in bank	11	12	8	9
Total items displaying DIF	5	6	3	6
Total number of thresholds	40	40	30	31
Total number of thresholds shared with 1+ country	25	25	16	11
Thresholds common to all groups	7	16	8	7
Percentage thresholds shared with 1 or more countries	63%	63%	54%	36%
Russia				
Total items in bank	11	12	8	9
Total items displaying DIF	5	3	1	2
Total number of thresholds	40	47	30	35
Total number of thresholds shared with 1+ country	26	39	20	7
Thresholds common to all groups	7	16	8	7
Percentage thresholds shared with 1 or more countries	65%	83%	67%	20%
Zimbabwe				
Total items in bank	9	12	7	9
Total items displaying DIF	6	2	3	7
Total number of thresholds	34	47	27	35
Thresholds common to all groups	7	16	8	7
Total number of thresholds shared with 1+ country	15	32	12	7
Percentage thresholds shared with 1 or more countries	44%	68%	44%	20%
India				
Total items in bank	11	12	8	9
Total items displaying DIF	6	5	3	6
Total number of thresholds	39	47	30	35
Thresholds common to all groups	7	16	8	7
Total number of thresholds shared with 1+ country	26	27	12	11
Percentage thresholds shared with 1 or more countries	67%	74%	40%	31%

**Table 2:** Summary of pairs of cultures showing DIF across all item bank items

Domain	Item	Wording	Countries
Physical	f1.4	To what extent do you feel that (physical) pain prevents you from doing what you need to do?	Zimbabwe and Russia
	f2.1	How easily do you get tired?	Zimbabwe and India
	f2.3	How satisfied are you with the energy that you have?	Zimbabwe and India
	f10.1	To what extent are you able to carry out your daily activities?	Zimbabwe and India
	f10.2	To what extent do you have difficulty in performing your routine activities?	All countries
	f10.4	How much are you bothered by any limitations in performing everyday living activities?	All four countries
	f12.2	Do you feel able to carry out your duties?	Zimbabwe and Russia
	f12.4	How satisfied are you with your capacity for work?	UK and all three others
Psychological	f4.1	How much do you enjoy life?	UK and all three others
	f4.3	How positive do you feel about the future?	Russia and India
	f5.3	How well are you able to concentrate?	UK and Russia
	f6.1	How much do you value yourself?	UK and Zimbabwe
	f6.2	How much confidence do you have in yourself?	UK and all three others
	f8.1	How often do you have negative feelings, such as blue mood, despair, anxiety, depression?	UK and all three others
	f8.2	How worried do you feel?	Zimbabwe and India
	f8.3	How much do any feelings of sadness or depression interfere with your everyday functioning?	UK and Zimbabwe
Social	f13.1	How alone do you feel in your life?	Zimbabwe and India
	f13.2	Do you feel happy about your relationship with your family members?	UK and Zimbabwe
	f13.4	How satisfied are you with your ability to provide for or support others?	All four countries
	f14.1	Do you get the kind of support from others that you need?	India and all three others
	f14.4	How satisfied are you with the support you get from your friends?	UK and all three others
	f15.3	How satisfied are you with your sex life?	All four countries
	f15.4	Are you bothered by any difficulties in your sex life?	All four countries
Environmental	f16.1	How safe do you feel in your daily life?	All four countries
	f16.4	How satisfied are you with your physical safety and security?	All four countries
	f17.3	How satisfied are you with the conditions of your living place?	All four countries
	f18.3	How satisfied are you with your financial situation?	All four countries
	f20.2	To what extent do you have opportunities for acquiring the information that you feel you need?	All four countries
	f20.4	How satisfied are you with your opportunities to learn new information?	Russia and Zimbabwe
	f21.2	How much are you able to relax and enjoy yourself?	Russian and all three others

All ANOVA values significant following Bonferroni correction for multiple comparisons

### Physical quality of life

Differential item functioning between cultures was apparent for 8 out of 11 items (*P* < 0.005, 11 comparisons). Details of DIF are summarized in Table 2 and displayed comprehensively in Online Appendix 1. After splitting for DIF, category threshold disordering was apparent for some items. For instance, items f1.4 and f10.2 were disordered for respondents from Zimbabwe and rescored 0-0-1-2-2, and item f2.1 needed to be rescored for India (0-1-2-2-3).

**Fig. 2:**
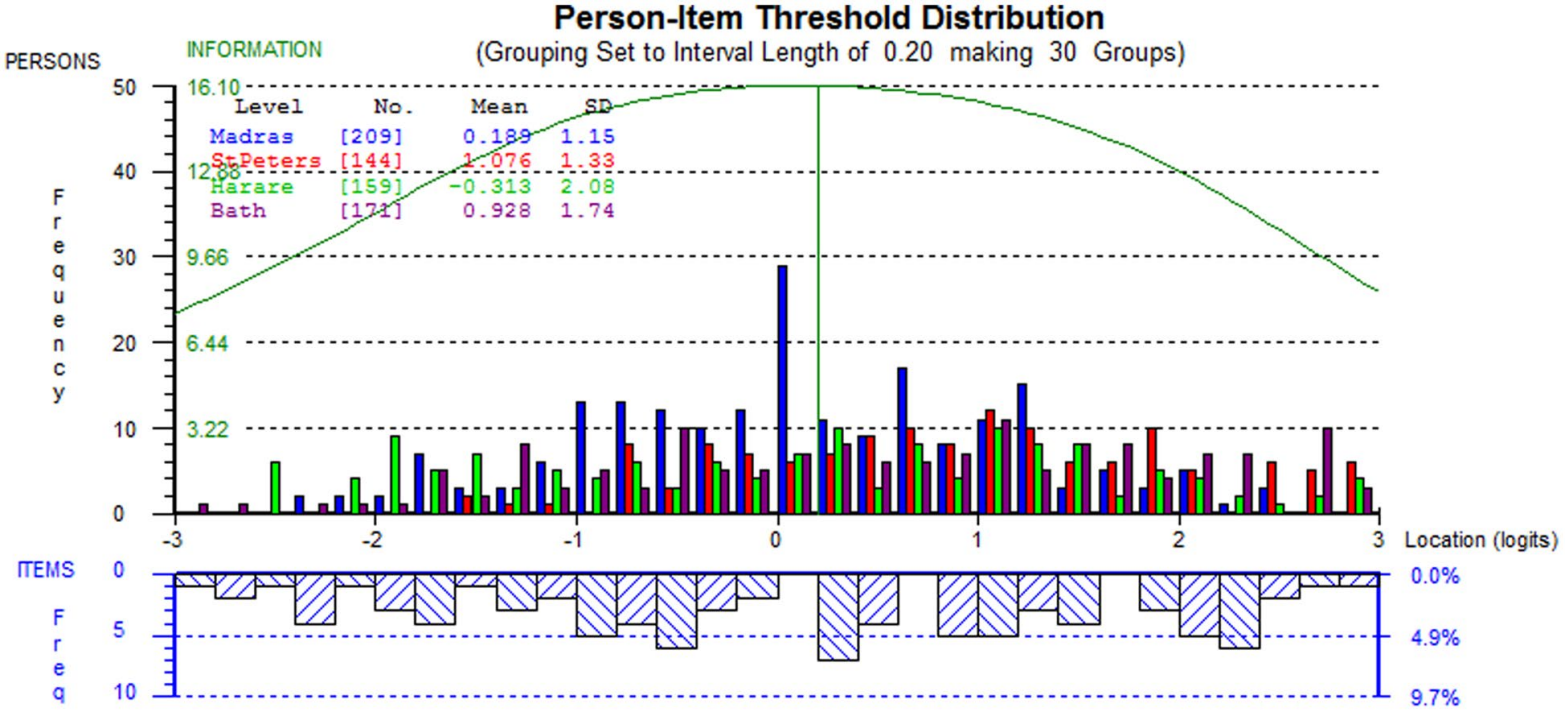
Comparison of person-item thresholds across all countries for the physical QOL domain. This figure compares with ‘location’ of participants on the underlying quality of life continuum from ± 3 logits (~ ± 3 standard deviations above and below the mean) shown above the *x*-axis and the ‘location’ of item information the same scale shown below the *x*-axis. As the item information covers a greater range of QoL than participants report, there are no floor or ceiling effects

After rescoring, items f1.4 and f9.3 misfit the Rasch model in the Zimbabwe sample, and they were removed from further analysis. After removing these items, no other violations of the Rasch model were apparent, and reliability was high (PSI = 0.92). Figure 2 shows an excellent spread of item information which covers a wide range of QoL (shown on the *x*-axis).

### Psychological quality of life

We observed significant ANOVA interactions for 8 of the 12 items in the psychological domain (*P* < 0.004, 12 comparisons). Detailed DIF results are presented in both Table 2 and Online Appendix 1. After splitting for DIF, two items required rescoring to compensate for their disordered thresholds. Item f8.3 was rescored for the UK and Zimbabwe samples (0-1-2-2-3), and item f6.1 was rescored for the Indian and Russian samples (0-1-1-2-3).

**Fig. 3:**
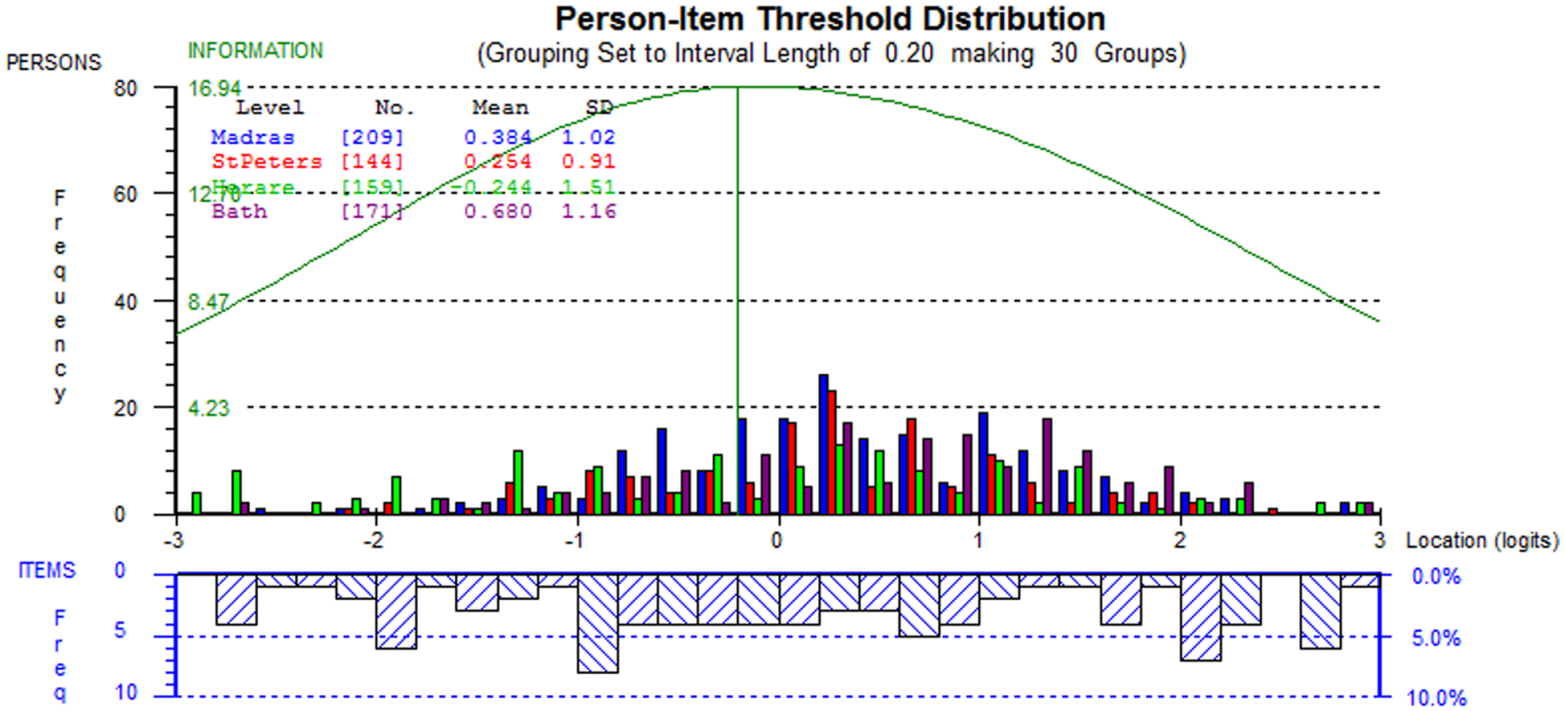
Comparison of person-item thresholds across all countries for the psychological QOL domain

Following splitting for DIF and rescoring, there were no other breaches of Rasch model assumptions, and reliability was high (PSI = 0.89) (Fig.3).

### Social quality of life

**Fig. 4:**
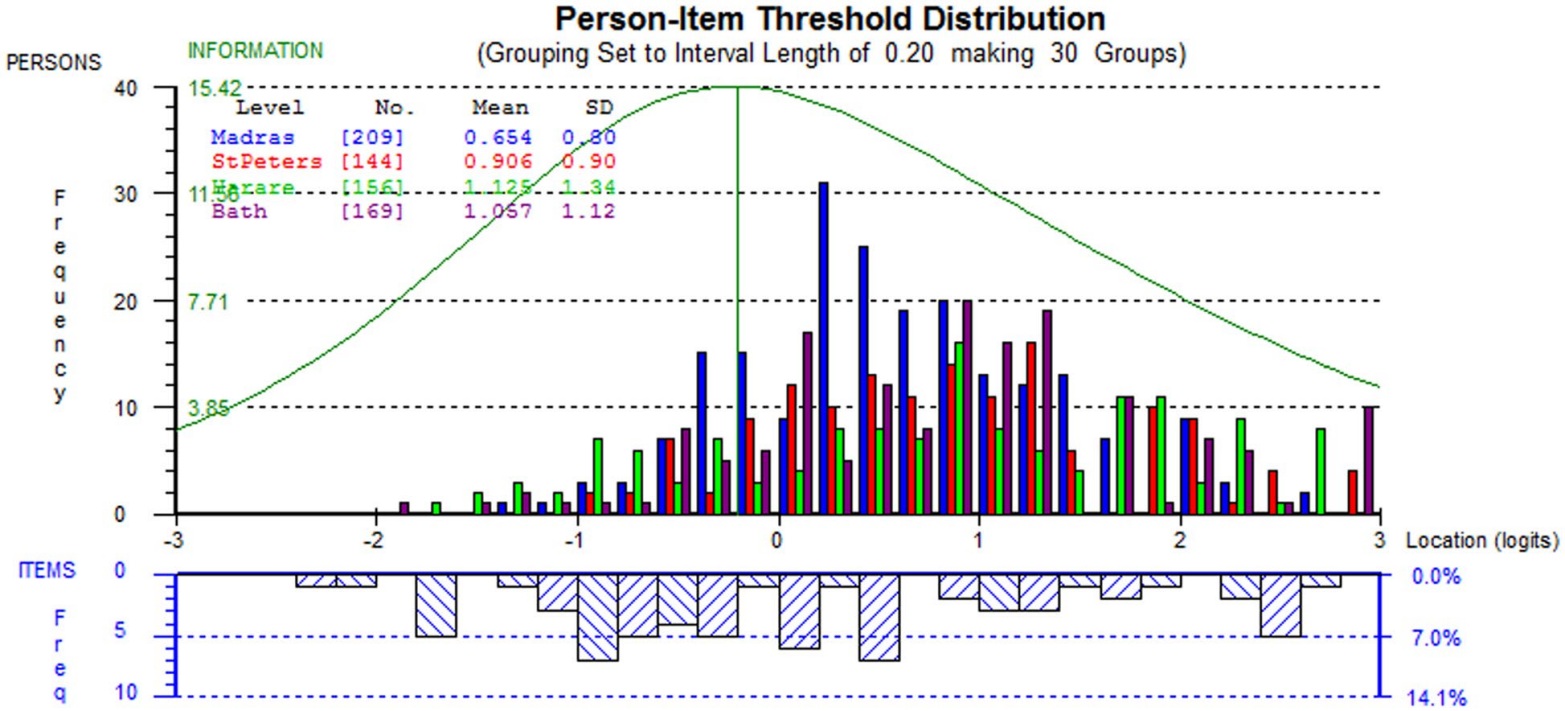
Comparison of person-item thresholds across all countries for the social QOL domain

**Fig. 5:**
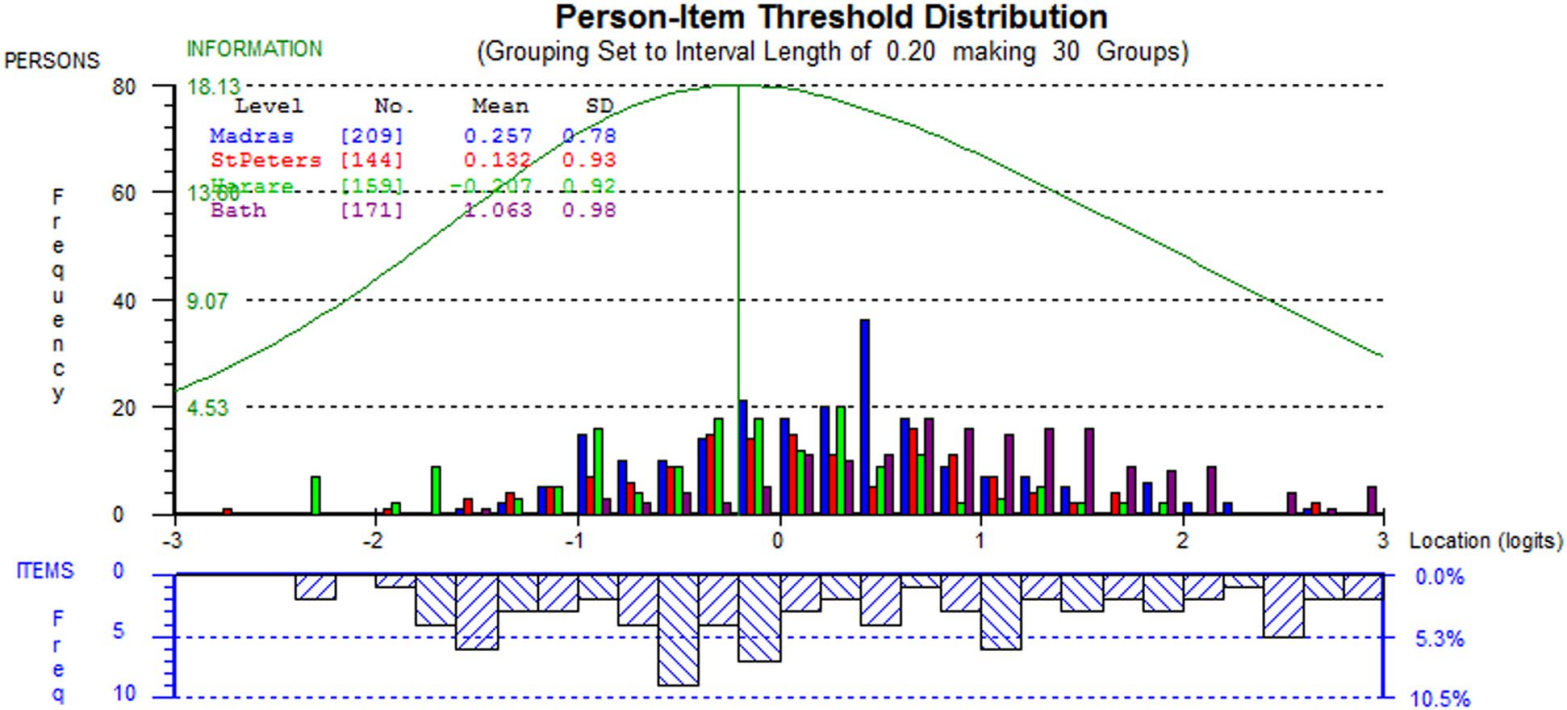
Comparison of person-item thresholds across all countries for the environmental QOL domain

ANOVA tests identified by-country DIF for seven items (*P* < 0.006, 8 comparisons; see Table 2 and Online Appendix 1). After splitting for DIF based on post hoc Tukey Tests, item f13.4 misfit the PCM and was removed from the Zimbabwean sample. Following this modification, there were no more breaches of IRT model assumptions. Reliability was acceptable, but rather low for the social quality of life items (mean PSI = 0.70). The information and targeting of the scale was good (see Figs. 4, 5), although there appeared to be a small ceiling effect for people from UK and Russia, a small proportion of whom (10 from the UK, 5 from Russia) fell outside the measurable range of the scale.

### Environmental QoL

ANOVA tests identified by-country DIF for 7 of the items in the item bank (*P* < 0.006, 9 comparisons; see Table 2 and Online Appendix 1). After splitting for DIF based on post hoc Tukey tests, item f18.3 misfit the PCM and was removed from the UK sample. Data from the three other countries showed good fit to the PCM after allowing item threshold parameters to vary by country (*P* > 0.01, see Table 3). Reliability was acceptable (mean PSI = 0.70).

**Table 3:** Psychometric summary for the four item banks

Centre	Item location		Person location		*χ* ^2^	*P*	Reliability (PSI)	Unidimensional *t* test (%)
	Mean	SD	Mean	SD				
Physical	0.083	2.31	0.52	1.55	160.56	0.001	0.92	6.85
Psychological	0.06	1.39	0.25	1.15	147.48	0.006	0.89	7.05
Social	− 0.09	2.3	0.74	1.05	131.09	0.003	0.78	5.60
Environmental	0.29	1.87	0.16	0.9	121.21	0.060	0.79	7.17

*PSI* Person separation index

### Simulated computerized adaptive testing

The item banks performed well compared to longer paper-based versions of the WHOQOL, creating measurement that was as reliable as the 26-item WHOQOL-BREF and the 100-item WHOQOL-100 with a mean of 12.5 and 18.5 items, respectively. This indicates that WHOQOL could be between 48 and 81% briefer than the existing paper versions in each of the four cultures. Details of the item parameters used in these simulations are provided in Online Appendix 2 (Table 4).

**Table 4:** Results of simulated computerized adaptive testing for each item bank in each of the four cultures

Scale	Domains	Original scale information		Stopping rule	CAT simulation (Bath bank)			CAT simulation (Harare bank)			CAT simulation (Madras bank)			CAT simulation (St. Petersburg bank)		
		No. of items	Reliability (alpha)	Reliability. matched standard error (SE)	Items (median)	Items (range)	Actual standard error (SE)	Items (median)	Items (range)	Actual standard (SE)	Items (median)	Items (range)	Actual standard error (SE)	Items (median)	Items (range)	Actual standard error (SE)
WHOQOL-BREF	Physical	7	0.82	0.42	4	4–8	0.4	4	4–7	039	4	4–7	0.4	4	4–7	0.4
	Psychological	6	0.81	0.44	4	3–6	0.42	4	3–6	0.41	4	3–6	0.42	4	3–6	0.42
	Social	3	0.68	0.55	3	3–7	0.53	2	2–7	0.53	2	2–4	0.5	2	2–4	0.5
	Environmental	8	0.8	0.45	4	3–7	0.43	3	2–7	0.43	3	2–7	0.43	3	2–7	0.43
WHOQOL-100*	Physical	16	0.86	0.37	6	5–11	0.35	6	5–10	0.36	6	5–11	0.35	6	5–11	0.35
	Physical	20	0.82	0.42	4	4–7	0.4	4	4–7	0.4	4	4–7	0.4	4	4–7	0.4
	Social	12	0.73	0.52	3	2–7	0.52	4	4–7	0.4.8	3	4–7	0.47	3	2–7	0.5
	Environmental	32	0.85	0.39	5	4–7	0.38	4	3–7	0.38	4	3–7	0.38	4	3–8	0.38

*WHOQOL-100 domains arranged into the same format as the WHOQOL-BREF

## Discussion

We found statistically significant DIF in many items in the 40-item WHOQOL item bank [16], in the four language versions assessed. We show that the process of DIF analysis provides useful cultural insights by highlighting how different items perform in different cultures, and allows data from all four countries to fit the Rasch model. We demonstrate how cross-cultural QoL assessment can be improved using item response theory, item anchoring, and computerized adaptive testing.

We highlight issues with cross-cultural DIF that have been demonstrated in WHOQOL measures elsewhere [3, 39], but the present study advances this field by applying an item anchoring solution which allows DIF to be accounted for, by permitting item bank parameters to vary between cultures. This method is attractive not only because it will improve the comparability of results from cross-cultural investigations, but also because it retains as many items as possible, which, in this case, have all been shown to be internationally valid [1, 2].

The method used in the present study has potential to be applied widely in QoL research. It is conceivable that the same techniques could be used to allow items with DIF which occurred between gender, age, or disease groups, to be calibrated on the same metric scale. An example would be facilitating QoL assessments that are both disease specific, because items could be included which were specific to single diseases, and generic across diseases, as estimates would be directly comparable across patient groups.

The techniques demonstrated here provide a framework for greater understanding of cultural differences in international quality of life research. For example, DIF analysis of the item “How much do you value yourself” demonstrated that Zimbabweans rate this item more highly than the four other cultures tested, suggesting that even when psychological QoL is poor, Zimbabweans value themselves more highly than people in the other cultures.

Similarly, DIF was present between Zimbabwe and India for the item “How satisfied are you with your financial situation?” Here, participants from India scored significantly higher than Zimbabweans at all levels of environmental QoL. These results are especially interesting in the context of economic data from the World Bank which shows that Zimbabweans had much higher Gross National Income in the years which the WHOQOL data were collected ($614 vs $416),^1^ which may reflect differences in culturally varying mediators between income and quality of life.

The study has some limitations. Although WHOQOL-100 data are available for 15 countries, we decided to develop this analysis using three randomly selected countries using an item bank which had previously been shown to work well, both in terms of fit to the Rasch model and an ability to produce adaptive assessments [16]. While the current study demonstrated that the linking approach was suitable for dealing with DIF between countries, it may not be applicable to every country. For example, if a country did not have sufficient shared thresholds to be adequately linked to the rest of the item bank, then it would be impossible to accurately link the banks.

Identification of DIF using the ANOVA method makes it vulnerable to sample size issues and at risk of identifying statistically significant, but non-substantive DIF between countries, which will naturally increase in line with growing sample sizes [6]. Over-identification of DIF is not necessarily problematic where items are ‘split’ for DIF and retained in the scale, rather than discarded, as they might be in a validation study which did not use adaptive testing or IRT scoring [28].

When conducting IRT analyses, a number of different models are available which estimate different parameters [40]. In the current study, we fitted our data to the partial credit model which estimates a single parameter for item threshold ‘difficulty’ (i.e. the level of QoL which is represented at the threshold between each of the Likert response categories). The widely used graded response model [41] estimates an additional parameter related to item discrimination (i.e. the extent to which item thresholds discriminate between different levels of underlying QoL). The parsimony of the Rasch model leads to a tendency to produce instruments with fewer items [42]. By retaining fewer items in a scale, there are necessarily fewer thresholds which may be used to anchor scales in which items have been split to accommodate DIF. In the current study, we found that a reasonable number of shared thresholds could be retained even when there was some DIF present for many of the items, although the number of thresholds used to link the environmental QoL scale was low (20%). We note that there is somewhat limited evidence relating to the number of common thresholds for linking QoL items with polytomous responses, and this would make productive area for future research.

Due to the lack of additional data on which to conduct CAT assessments, we used simulated data based on a wide range of QoL. Due to the simulated nature of the data, it is possible that ‘real-world’ assessments using CAT may be less efficient, especially in instances when a person’s responses differ substantially from those expected by the model. With larger sample sizes, greater confidence in the results would have been established by successfully cross-validating the psychometric models.

We acknowledge a growing body of literature which assesses the practical impact of DIF beyond statistical significance. Results of these studies are mixed, and while some demonstrate both clinically and statistically significant differences in scores at the group level [25], others indicate that the effect of DIF on group level comparisons was negligible [43]. We did not replicate such analyses in the current study for several reasons. Firstly, remediation of DIF using the item-splitting solution will improve measurement regardless of demonstrable differences in scores at the group level, and, as we have demonstrated, can be implemented easily without sacrificing items. Secondly, while we acknowledge that DIF may or may not have an impact on scale total scores, there is some uncertainty as to the impact of DIF on item selection during CAT assessment.

In summary, we demonstrate the application of a method that can simultaneously increase understanding of cross-cultural QoL, and improve its estimation using questionnaire scales. By allowing the calibration of item parameters to vary across countries, it was possible to create measurement which was valid both within and between cultures, alongside item banks that were suitable for computerized adaptive testing.

## Electronic supplementary material

Below is the link to the electronic supplementary material.

Supplementary material 1 (DOCX 560 KB)

Supplementary material 2 (DOCX 22 KB)

## Footnotes

1 1995−1998. GNI Data accessed from on 14/07/2015. https://data.worldbank.org/indicator/NY.GDP.PCAP.CD?page=3.
