# Supplementary material for: Adjusting for cross-cultural differences in computer-adaptive tests of quality of life
Source: Qual Life Res. 2017 Dec 4;27(4):1027–39. doi: 10.1007/s11136-017-1738-7 (PMC5874271; doi:10.1007/s11136-017-1738-7)
Supplement: Supplementary file 1 — Supplementary material 1 (DOCX 560 KB) [file 11136_2017_1738_MOESM1_ESM.docx]

Gibbons and Skevington (2017). Adjusting for cross-cultural differences in international item banks for quality of life. Appendix 1 – Detailed differential item functioning results

Differential item functioning plots and post-doc Tukey test results for Item F1.4


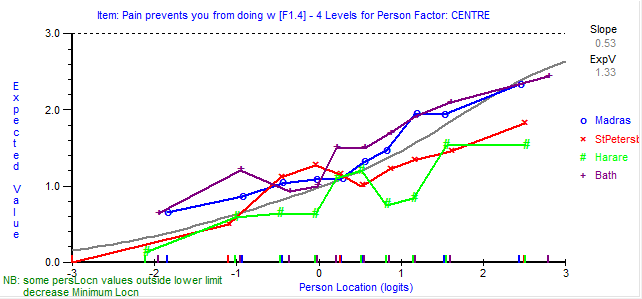


This figure shows the expected response to the item (*y-*axis) at each level across the continuum of quality of life (*x*-axis). Each country is plotted separately.

| Country 1 | Country 2 | Mean 1 | Mean 2 | Absolute Difference | Dmin criterion |
| --- | --- | --- | --- | --- | --- |
| Bath | Harare | 0.31 | -0.51 | 0.82 | 0.27 |
| Bath | StPete | 0.31 | -0.26 | 0.57 | 0.27 |
| Bath | Madras | 0.31 | 0.24 | 0.07 | 0.25 |
| StPete | Madras | -0.26 | 0.24 | 0.5 | 0.26 |
| Harare | Madras | -0.51 | 0.24 | 0.75 | 0.25 |
| Harare | StPete | -0.51 | -0.26 | 0.25 | 0.28 |

DIF is evident when the absolute difference is greater than the Dmin criterion.

Differential item functioning plots and post-doc Tukey test results for Item F2.1


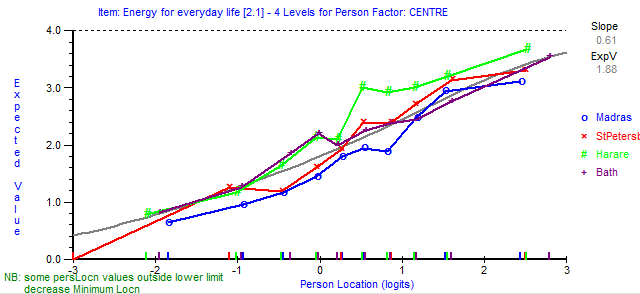


This figure shows the expected response to the item (*y-*axis) at each level across the continuum of quality of life (*x*-axis). Each country is plotted separately.

| Country 1 | Country 2 | Mean 1 | Mean 2 | Absolute Difference | Dmin criterion |
| --- | --- | --- | --- | --- | --- |
| Bath | Harare | 0.07 | 0.32 | 0.25 | 0.23 |
| Bath | StPete | 0.07 | 0.05 | 0.02 | 0.23 |
| Bath | Madras | 0.07 | -0.31 | 0.38 | 0.21 |
| StPete | Madras | 0.05 | -0.31 | 0.36 | 0.22 |
| Harare | Madras | 0.32 | -0.31 | 0.63 | 0.22 |
| Harare | StPete | 0.32 | 0.05 | 0.27 | 0.24 |

DIF is evident when the absolute difference is greater than the Dmin criterion.

Differential item functioning plots and post-doc Tukey test results for Item F2.3


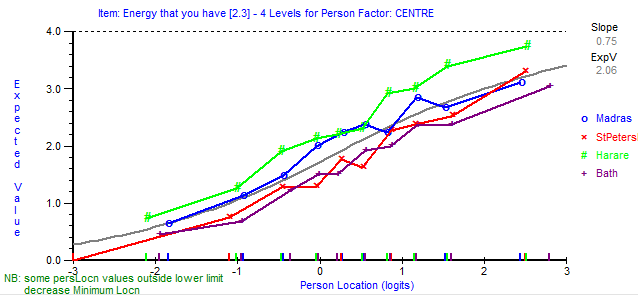


This figure shows the expected response to the item (*y-*axis) at each level across the continuum of quality of life (*x*-axis). Each country is plotted separately.

| Country 1 | Country 2 | Mean 1 | Mean 2 | Absolute Difference | Dmin criterion |
| --- | --- | --- | --- | --- | --- |
| Bath | Harare | -0.4 | 0.47 | 0.87 | 0.25 |
| Bath | StPete | -0.4 | -0.21 | 0.19 | 0.25 |
| Bath | Madras | -0.4 | 0.13 | 0.53 | 0.23 |
| StPete | Madras | -0.21 | 0.13 | 0.34 | 0.24 |
| Harare | Madras | 0.47 | 0.13 | 0.34 | 0.23 |
| Harare | StPete | 0.47 | -0.21 | 0.68 | 0.26 |

DIF is evident when the absolute difference is greater than the Dmin criterion.

Differential item functioning plots and post-doc Tukey test results for Item F10.1


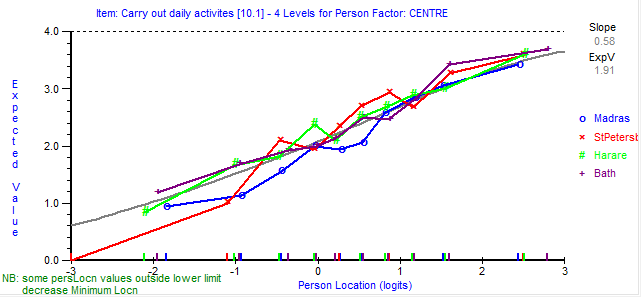


This figure shows the expected response to the item (*y-*axis) at each level across the continuum of quality of life (*x*-axis). Each country is plotted separately.

| Country 1 | Country 2 | Mean 1 | Mean 2 | Absolute Difference | Dmin criterion |
| --- | --- | --- | --- | --- | --- |
| Bath | Harare | 0.16 | 0.01 | 0.15 | 0.23 |
| Bath | StPetes | 0.16 | 0.17 | 0.01 | 0.24 |
| Bath | Madras | 0.16 | -0.22 | 0.38 | 0.22 |
| StPetes | Madras | 0.17 | -0.22 | 0.39 | 0.23 |
| Harare | Madras | 0.01 | -0.22 | 0.23 | 0.22 |
| Harare | StPetes | 0.01 | 0.17 | 0.16 | 0.24 |

DIF is evident when the absolute difference is greater than the Dmin criterion.

Differential item functioning plots and post-doc Tukey test results for Item F10.2


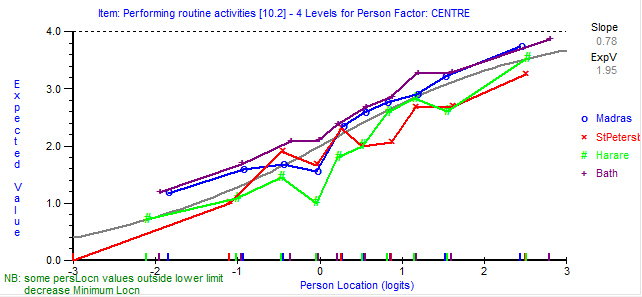


This figure shows the expected response to the item (*y-*axis) at each level across the continuum of quality of life (*x*-axis). Each country is plotted separately.

| Country 1 | Country 2 | Mean 1 | Mean 2 | Absolute Difference | Dmin criterion |
| --- | --- | --- | --- | --- | --- |
| Bath | Harare | 0.39 | -0.23 | 0.62 | 0.28 |
| Bath | StPetes | 0.39 | -0.33 | 0.72 | 0.28 |
| Bath | Madras | 0.39 | 0.14 | 0.25 | 0.26 |
| StPetes | Madras | -0.33 | 0.14 | 0.47 | 0.27 |
| Harare | Madras | -0.23 | 0.14 | 0.37 | 0.26 |
| Harare | StPetes | -0.23 | -0.33 | 0.1 | 0.29 |

DIF is evident when the absolute difference is greater than the Dmin criterion.

Differential item functioning plots and post-doc Tukey test results for Item F10.4


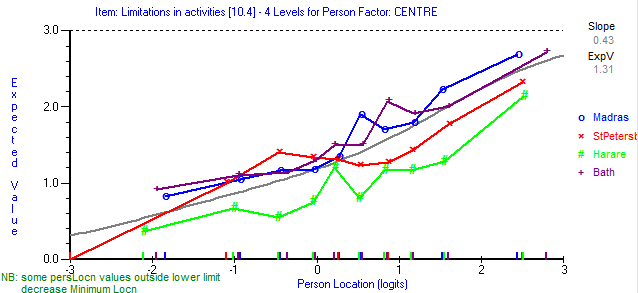


This figure shows the expected response to the item (*y-*axis) at each level across the continuum of quality of life (*x*-axis). Each country is plotted separately.

| Country 1 | Country 2 | Mean 1 | Mean 2 | Absolute Difference | Dmin criterion |
| --- | --- | --- | --- | --- | --- |
| Bath | Harare | 0.27 | -0.56 | 0.83 | 0.28 |
| Bath | StPetes | 0.27 | -0.12 | 0.39 | 0.28 |
| Bath | Madras | 0.27 | 0.24 | 0.03 | 0.26 |
| StPetes | Madras | -0.12 | 0.24 | 0.36 | 0.27 |
| Harare | Madras | -0.56 | 0.24 | 0.8 | 0.26 |
| Harare | StPetes | -0.56 | -0.12 | 0.44 | 0.29 |

DIF is evident when the absolute difference is greater than the Dmin criterion.

Differential item functioning plots and post-doc Tukey test results for Item F12.2


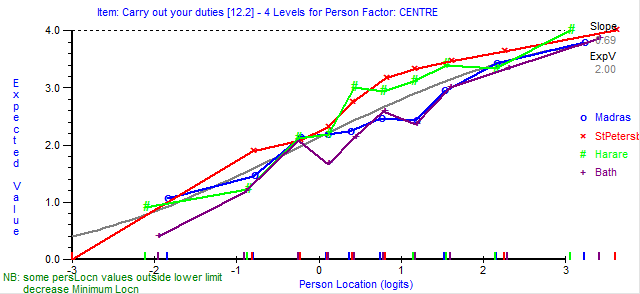


This figure shows the expected response to the item (*y-*axis) at each level across the continuum of quality of life (*x*-axis). Each country is plotted separately.

| Country 1 | Country 2 | Mean 1 | Mean 2 | Absolute Difference | Dmin criterion |
| --- | --- | --- | --- | --- | --- |
| Bath | Harare | -0.25 | 0.15 | 0.4 | 0.26 |
| Bath | StPetes | -0.25 | 0.38 | 0.63 | 0.26 |
| Bath | Madras | -0.25 | -0.12 | 0.13 | 0.24 |
| StPetes | Madras | 0.38 | -0.12 | 0.5 | 0.25 |
| Harare | Madras | 0.15 | -0.12 | 0.27 | 0.24 |
| Harare | StPetes | 0.15 | 0.38 | 0.23 | 0.27 |

DIF is evident when the absolute difference is greater than the Dmin criterion.

Differential item functioning plots and post-doc Tukey test results for Item F12.4


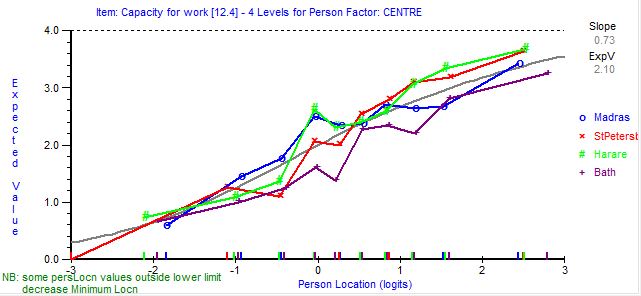


This figure shows the expected response to the item (*y-*axis) at each level across the continuum of quality of life (*x*-axis). Each country is plotted separately.

| Country 1 | Country 2 | Mean 1 | Mean 2 | Absolute Difference | Dmin criterion |
| --- | --- | --- | --- | --- | --- |
| Bath | Harare | -0.37 | 0.19 | 0.56 | 0.28 |
| Bath | StPetes | -0.37 | 0.19 | 0.56 | 0.29 |
| Bath | Madras | -0.37 | 0.08 | 0.45 | 0.26 |
| StPetes | Madras | 0.19 | 0.08 | 0.11 | 0.27 |
| Harare | Madras | 0.19 | 0.08 | 0.11 | 0.26 |
| Harare | StPetes | 0.19 | 0.19 | 0 | 0.29 |

DIF is evident when the absolute difference is greater than the Dmin criterion.

Differential item functioning plots and post-doc Tukey test results for Item F4.1


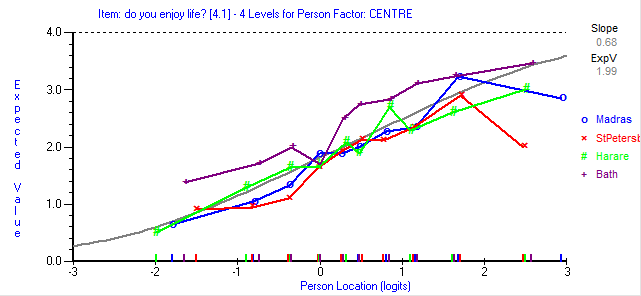


This figure shows the expected response to the item (*y-*axis) at each level across the continuum of quality of life (*x*-axis). Each country is plotted separately.

| Country 1 | Country 2 | Mean 1 | Mean 2 | Absolute Difference | Dmin criterion |
| --- | --- | --- | --- | --- | --- |
| Bath | Harare | 0.47 | -0.14 | 0.61 | 0.24 |
| Bath | StPete | 0.47 | -0.22 | 0.69 | 0.25 |
| Bath | Madras | 0.47 | -0.14 | 0.61 | 0.23 |
| StPete | Madras | -0.22 | -0.14 | 0.08 | 0.25 |
| Harare | Madras | -0.14 | -0.14 | 0 | 0.24 |
| Harare | StPete | -0.14 | -0.22 | 0.08 | 0.27 |

DIF is evident when the absolute difference is greater than the Dmin criterion.

Differential item functioning plots and post-doc Tukey test results for Item F4.3


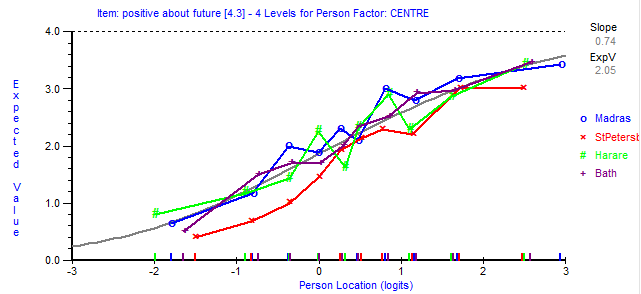


This figure shows the expected response to the item (*y-*axis) at each level across the continuum of quality of life (*x*-axis). Each country is plotted separately.

| Country 1 | Country 2 | Mean 1 | Mean 2 | Absolute Difference | Dmin criterion |
| --- | --- | --- | --- | --- | --- |
| Bath | Harare | 0.05 | 0.06 | 0.01 | 0.26 |
| Bath | StPete | 0.05 | -0.38 | 0.43 | 0.27 |
| Bath | Madras | 0.05 | 0.18 | 0.13 | 0.24 |
| StPete | Madras | -0.38 | 0.18 | 0.56 | 0.25 |
| Harare | Madras | 0.06 | 0.18 | 0.12 | 0.25 |
| Harare | StPete | 0.06 | -0.38 | 0.44 | 0.27 |

DIF is evident when the absolute difference is greater than the Dmin criterion.

Differential item functioning plots and post-doc Tukey test results for Item F5.3


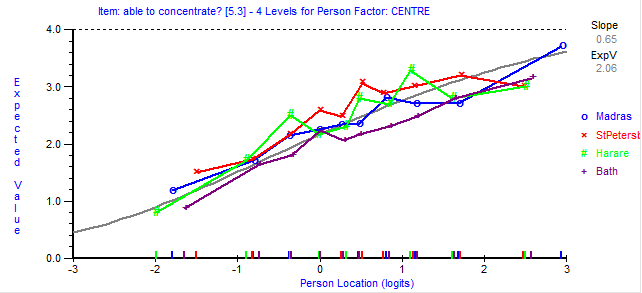


This figure shows the expected response to the item (*y-*axis) at each level across the continuum of quality of life (*x*-axis). Each country is plotted separately.

| Country 1 | Country 2 | Mean 1 | Mean 2 | Absolute Difference | Dmin criterion |
| --- | --- | --- | --- | --- | --- |
| Bath | Harare | -0.36 | 0.03 | 0.39 | 0.29 |
| Bath | StPetes | -0.36 | 0.33 | 0.69 | 0.30 |
| Bath | Madras | -0.36 | 0.03 | 0.39 | 0.27 |
| StPetes | Madras | 0.33 | 0.03 | 0.3 | 0.28 |
| Harare | Madras | 0.03 | 0.03 | 0 | 0.27 |
| Harare | StPetes | 0.03 | 0.33 | 0.3 | 0.30 |

DIF is evident when the absolute difference is greater than the Dmin criterion.

Differential item functioning plots and post-doc Tukey test results for Item F6.1


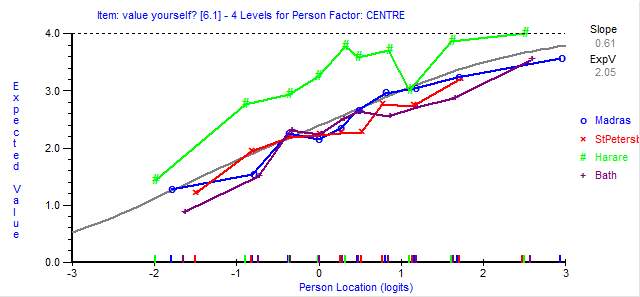


This figure shows the expected response to the item (*y-*axis) at each level across the continuum of quality of life (*x*-axis). Each country is plotted separately.

| Country 1 | Country 2 | Mean 1 | Mean 2 | Absolute Difference | Dmin criterion |
| --- | --- | --- | --- | --- | --- |
| Bath | Harare | -0.39 | 0.83 | 1.22 | 0.26 |
| Bath | StPetes | -0.39 | -0.25 | 0.14 | 0.26 |
| Bath | Madras | -0.39 | -0.14 | 0.25 | 0.24 |
| StPetes | Madras | -0.25 | -0.14 | 0.11 | 0.25 |
| Harare | Madras | 0.83 | -0.14 | 0.97 | 0.24 |
| Harare | StPetes | 0.83 | -0.25 | 1.08 | 0.27 |

DIF is evident when the absolute difference is greater than the Dmin criterion.

Differential item functioning plots and post-doc Tukey test results for Item F6.2


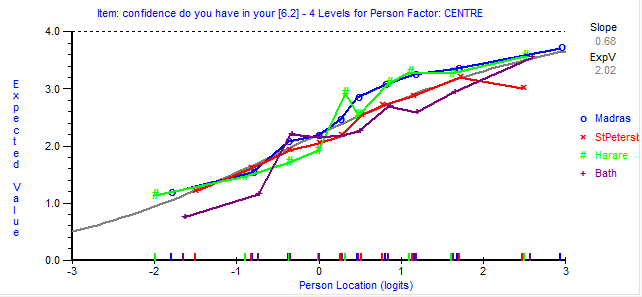


This figure shows the expected response to the item (*y-*axis) at each level across the continuum of quality of life (*x*-axis). Each country is plotted separately.

| Country 1 | Country 2 | Mean 1 | Mean 2 | Absolute Difference | Dmin criterion |
| --- | --- | --- | --- | --- | --- |
| Bath | Harare | -0.25 | 0.13 | 0.38 | 0.26 |
| Bath | StPetes | -0.25 | -0.09 | 0.16 | 0.27 |
| Bath | Madras | -0.25 | 0.2 | 0.45 | 0.25 |
| StPetes | Madras | -0.09 | 0.2 | 0.29 | 0.26 |
| Harare | Madras | 0.13 | 0.2 | 0.07 | 0.25 |
| Harare | StPetes | 0.13 | -0.09 | 0.22 | 0.27 |

DIF is evident when the absolute difference is greater than the Dmin criterion.

Differential item functioning plots and post-doc Tukey test results for Item F8.1


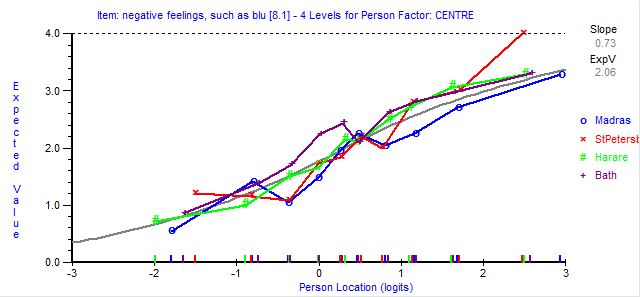


This figure shows the expected response to the item (*y-*axis) at each level across the continuum of quality of life (*x*-axis). Each country is plotted separately.

| Country 1 | Country 2 | Mean 1 | Mean 2 | Absolute Difference | Dmin criterion |
| --- | --- | --- | --- | --- | --- |
| Bath | Harare | 0.26 | 0.05 | 0.21 | 0.27 |
| Bath | StPetes | 0.26 | -0.03 | 0.29 | 0.27 |
| Bath | Madras | 0.26 | -0.2 | 0.46 | 0.25 |
| StPetes | Madras | -0.03 | -0.2 | 0.17 | 0.26 |
| Harare | Madras | 0.05 | -0.2 | 0.25 | 0.25 |
| Harare | StPetes | 0.05 | -0.03 | 0.08 | 0.28 |

DIF is evident when the absolute difference is greater than the Dmin criterion.

Differential item functioning plots and post-doc Tukey test results for Item F8.2


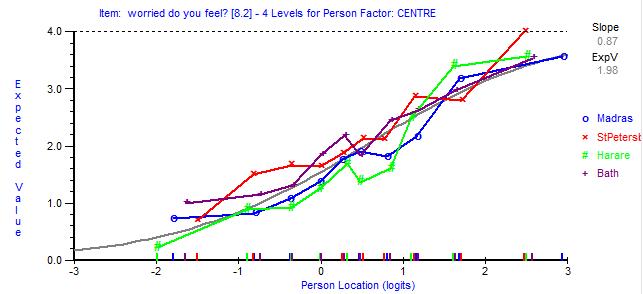


This figure shows the expected response to the item (*y-*axis) at each level across the continuum of quality of life (*x*-axis). Each country is plotted separately.

| Country 1 | Country 2 | Mean 1 | Mean 2 | Absolute Difference | Dmin criterion |
| --- | --- | --- | --- | --- | --- |
| Bath | Harare | 0.17 | -0.22 | 0.39 | 0.26 |
| Bath | StPetes | 0.17 | 0.21 | 0.04 | 0.27 |
| Bath | Madras | 0.17 | -0.13 | 0.3 | 0.25 |
| StPetes | Madras | 0.21 | -0.13 | 0.34 | 0.26 |
| Harare | Madras | -0.22 | -0.13 | 0.09 | 0.25 |
| Harare | StPetes | -0.22 | 0.21 | 0.43 | 0.28 |

DIF is evident when the absolute difference is greater than the Dmin criterion.

Differential item functioning plots and post-doc Tukey test results for Item F8.3


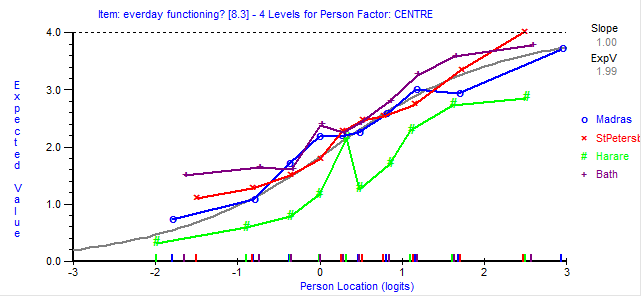


This figure shows the expected response to the item (*y-*axis) at each level across the continuum of quality of life (*x*-axis). Each country is plotted separately.

| Country 1 | Country 2 | Mean 1 | Mean 2 | Absolute Difference | Dmin criterion |
| --- | --- | --- | --- | --- | --- |
| Bath | Harare | 0.37 | -0.58 | 0.95 | 0.27 |
| Bath | StPetes | 0.37 | 0.09 | 0.28 | 0.25 |
| Bath | Madras | 0.37 | 0.08 | 0.29 | 0.25 |
| StPetes | Madras | 0.09 | 0.08 | 0.01 | 0.26 |
| Harare | Madras | -0.58 | 0.08 | 0.66 | 0.25 |
| Harare | StPetes | -0.58 | 0.09 | 0.67 | 0.28 |

DIF is evident when the absolute difference is greater than the Dmin criterion.

Differential item functioning plots and post-doc Tukey test results for Item F13.1


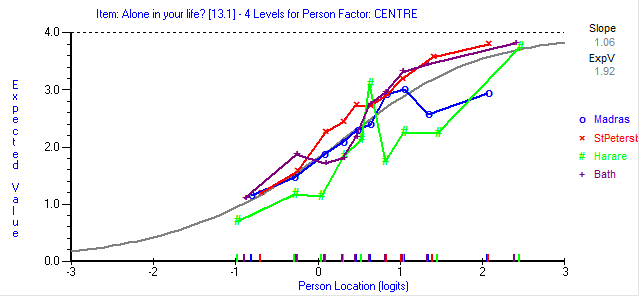


This figure shows the expected response to the item (*y-*axis) at each level across the continuum of quality of life (*x*-axis). Each country is plotted separately.

| Country 1 | Country 2 | Mean 1 | Mean 2 | Absolute Difference | Dmin criterion |
| --- | --- | --- | --- | --- | --- |
| Bath | Harare | 0.2 | -0.25 | 0.45 | 0.26 |
| Bath | StPetes | 0.2 | 0.27 | 0.07 | 0.26 |
| Bath | Madras | 0.2 | -0.11 | 0.31 | 0.24 |
| StPetes | Madras | 0.27 | -0.11 | 0.38 | 0.25 |
| Harare | Madras | -0.25 | -0.11 | 0.14 | 0.24 |
| Harare | StPetes | -0.25 | 0.27 | 0.52 | 0.27 |

DIF is evident when the absolute difference is greater than the Dmin criterion.

Differential item functioning plots and post-doc Tukey test results for Item F13.2


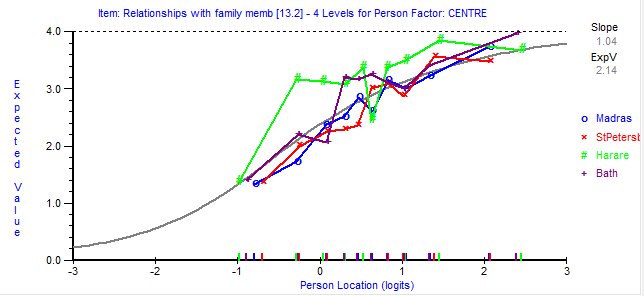


This figure shows the expected response to the item (*y-*axis) at each level across the continuum of quality of life (*x*-axis). Each country is plotted separately.

| Country 1 | Country 2 | Mean 1 | Mean 2 | Absolute Difference | Dmin criterion |
| --- | --- | --- | --- | --- | --- |
| Bath | Harare | 0.19 | 0.29 | 0.1 | 0.26 |
| Bath | StPetes | 0.19 | -0.13 | 0.32 | 0.27 |
| Bath | Madras | 0.19 | -0.11 | 0.3 | 0.24 |
| StPetes | Madras | -0.13 | -0.11 | 0.02 | 0.25 |
| Harare | Madras | 0.29 | -0.11 | 0.4 | 0.25 |
| Harare | StPetes | 0.29 | -0.13 | 0.42 | 0.27 |

DIF is evident when the absolute difference is greater than the Dmin criterion.

Differential item functioning plots and post-doc Tukey test results for Item F13.4


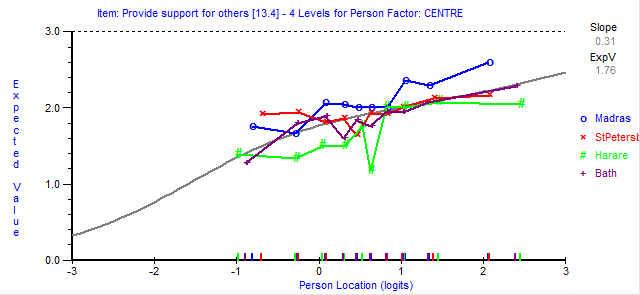


This figure shows the expected response to the item (*y-*axis) at each level across the continuum of quality of life (*x*-axis). Each country is plotted separately.

| Country 1 | Country 2 | Mean 1 | Mean 2 | Absolute Difference | Dmin criterion |
| --- | --- | --- | --- | --- | --- |
| Bath | Harare | -0.05 | -0.43 | 0.38 | 0.31 |
| Bath | StPetes | -0.05 | 0.03 | 0.08 | 0.32 |
| Bath | Madras | -0.05 | 0.36 | 0.41 | 0.29 |
| StPetes | Madras | 0.03 | 0.36 | 0.33 | 0.30 |
| Harare | Madras | -0.43 | 0.36 | 0.79 | 0.29 |
| Harare | StPetes | -0.43 | 0.03 | 0.46 | 0.32 |

DIF is evident when the absolute difference is greater than the Dmin criterion.

Differential item functioning plots and post-doc Tukey test results for Item F14.1


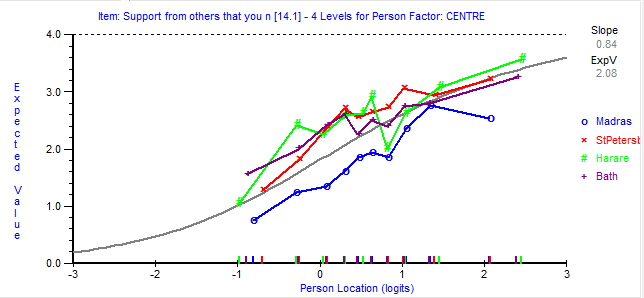


This figure shows the expected response to the item (*y-*axis) at each level across the continuum of quality of life (*x*-axis). Each country is plotted separately.

| Country 1 | Country 2 | Mean 1 | Mean 2 | Absolute Difference | Dmin criterion |
| --- | --- | --- | --- | --- | --- |
| Bath | Harare | 0.13 | 0.29 | 0.16 | 0.27 |
| Bath | StPetes | 0.13 | 0.31 | 0.18 | 0.28 |
| Bath | Madras | 0.13 | -0.47 | 0.6 | 0.25 |
| StPetes | Madras | 0.31 | -0.47 | 0.78 | 0.28 |
| Harare | Madras | 0.29 | -0.47 | 0.76 | 0.27 |
| Harare | StPetes | 0.29 | 0.31 | 0.02 | 0.29 |

DIF is evident when the absolute difference is greater than the Dmin criterion.

Differential item functioning plots and post-doc Tukey test results for Item F14.4


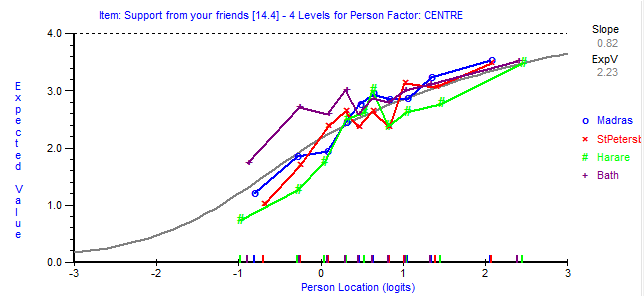


This figure shows the expected response to the item (*y-*axis) at each level across the continuum of quality of life (*x*-axis). Each country is plotted separately.

| Country 1 | Country 2 | Mean 1 | Mean 2 | Absolute Difference | Dmin criterion |
| --- | --- | --- | --- | --- | --- |
| Bath | Harare | 0.25 | -0.18 | 0.43 | 0.27 |
| Bath | StPetes | 0.25 | -0.01 | 0.26 | 0.27 |
| Bath | Madras | 0.25 | 0.05 | 0.2 | 0.25 |
| StPetes | Madras | -0.01 | 0.05 | 0.06 | 0.27 |
| Harare | Madras | -0.18 | 0.05 | 0.23 | 0.27 |
| Harare | StPetes | -0.18 | -0.01 | 0.17 | 0.29 |

DIF is evident when the absolute difference is greater than the Dmin criterion.

Differential item functioning plots and post-doc Tukey test results for Item F15.3


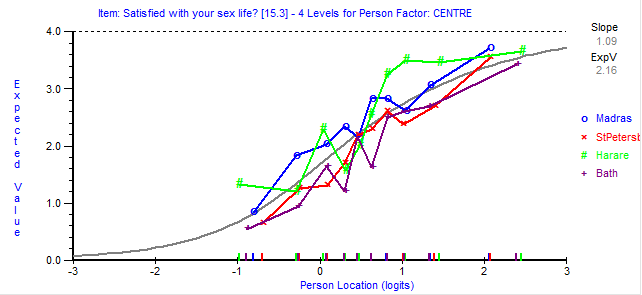


This figure shows the expected response to the item (*y-*axis) at each level across the continuum of quality of life (*x*-axis). Each country is plotted separately.

| Country 1 | Country 2 | Mean 1 | Mean 2 | Absolute Difference | Dmin criterion |
| --- | --- | --- | --- | --- | --- |
| Bath | Harare | -0.24 | 0.33 | 0.57 | 0.32 |
| Bath | StPetes | -0.24 | -0.19 | 0.05 | 0.31 |
| Bath | Madras | -0.24 | 0.22 | 0.46 | 0.29 |
| StPetes | Madras | -0.19 | 0.22 | 0.41 | 0.30 |
| Harare | Madras | 0.33 | 0.22 | 0.11 | 0.31 |
| Harare | StPetes | 0.33 | -0.19 | 0.52 | 0.33 |

DIF is evident when the absolute difference is greater than the Dmin criterion.

Differential item functioning plots and post-doc Tukey test results for Item F15.4


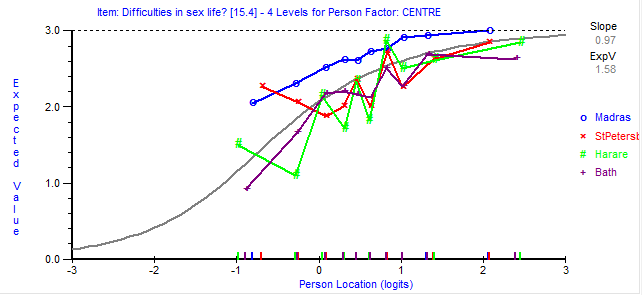


This figure shows the expected response to the item (*y-*axis) at each level across the continuum of quality of life (*x*-axis). Each country is plotted separately.

| Country 1 | Country 2 | Mean 1 | Mean 2 | Absolute Difference | Dmin criterion |
| --- | --- | --- | --- | --- | --- |
| Bath | Harare | -0.32 | -0.14 | 0.18 | 0.24 |
| Bath | StPetes | -0.32 | -0.1 | 0.22 | 0.25 |
| Bath | Madras | -0.32 | 0.47 | 0.79 | 0.23 |
| StPetes | Madras | -0.1 | 0.47 | 0.57 | 0.25 |
| Harare | Madras | -0.14 | 0.47 | 0.61 | 0.24 |
| Harare | StPetes | -0.14 | -0.1 | 0.04 | 0.27 |

DIF is evident when the absolute difference is greater than the Dmin criterion.

Differential item functioning plots and post-doc Tukey test results for Item F16.1


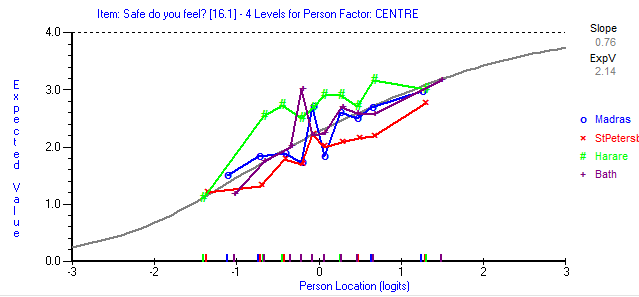


This figure shows the expected response to the item (*y-*axis) at each level across the continuum of quality of life (*x*-axis). Each country is plotted separately.

| Country 1 | Country 2 | Mean 1 | Mean 2 | Absolute Difference | Dmin criterion |
| --- | --- | --- | --- | --- | --- |
| Bath | Harare | 0 | 0.38 | 0.38 | 0.27 |
| Bath | StPetes | 0 | -0.35 | 0.35 | 0.27 |
| Bath | Madras | 0 | -0.01 | 0.01 | 0.25 |
| StPetes | Madras | -0.35 | -0.01 | 0.34 | 0.26 |
| Harare | Madras | 0.38 | -0.01 | 0.39 | 0.25 |
| Harare | StPetes | 0.38 | -0.35 | 0.73 | 0.28 |

DIF is evident when the absolute difference is greater than the Dmin criterion.

Differential item functioning plots and post-doc Tukey test results for Item F16.4


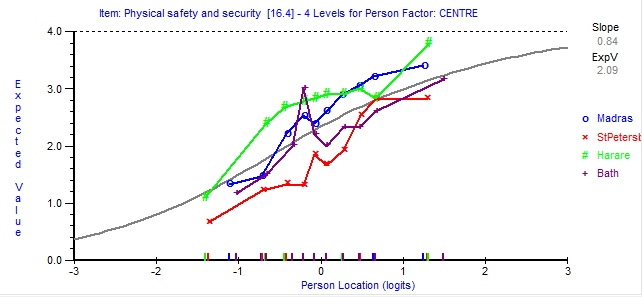


This figure shows the expected response to the item (*y-*axis) at each level across the continuum of quality of life (*x*-axis). Each country is plotted separately.

| Country 1 | Country 2 | Mean 1 | Mean 2 | Absolute Difference | Dmin criterion |
| --- | --- | --- | --- | --- | --- |
| Bath | Harare | -0.18 | 0.42 | 0.6 | 0.23 |
| Bath | StPetes | -0.18 | -0.56 | 0.38 | 0.23 |
| Bath | Madras | -0.18 | 0.29 | 0.47 | 0.21 |
| StPetes | Madras | -0.56 | 0.29 | 0.85 | 0.22 |
| Harare | Madras | 0.42 | 0.29 | 0.13 | 0.22 |
| Harare | StPetes | 0.42 | -0.56 | 0.98 | 0.24 |

DIF is evident when the absolute difference is greater than the Dmin criterion.

Differential item functioning plots and post-doc Tukey test results for Item F17.3


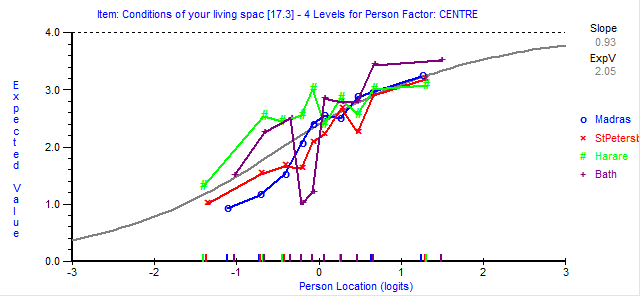


This figure shows the expected response to the item (*y-*axis) at each level across the continuum of quality of life (*x*-axis). Each country is plotted separately.

| Country 1 | Country 2 | Mean 1 | Mean 2 | Absolute Difference | Dmin criterion |
| --- | --- | --- | --- | --- | --- |
| Bath | Harare | 0.27 | 0.25 | 0.02 | 0.24 |
| Bath | StPetes | 0.27 | -0.27 | 0.54 | 0.25 |
| Bath | Madras | 0.27 | -0.14 | 0.41 | 0.23 |
| StPetes | Madras | -0.27 | -0.14 | 0.13 | 0.24 |
| Harare | Madras | 0.25 | -0.14 | 0.39 | 0.23 |
| Harare | StPetes | 0.25 | -0.27 | 0.52 | 0.25 |

DIF is evident when the absolute difference is greater than the Dmin criterion.

Differential item functioning plots and post-doc Tukey test results for Item F18.3


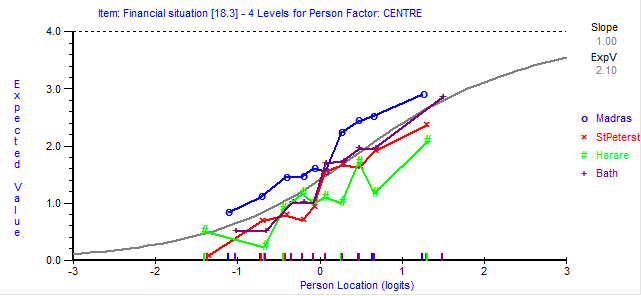


This figure shows the expected response to the item (*y-*axis) at each level across the continuum of quality of life (*x*-axis). Each country is plotted separately.

| Country 1 | Country 2 | Mean 1 | Mean 2 | Absolute Difference | Dmin criterion |
| --- | --- | --- | --- | --- | --- |
| Bath | Harare | 0.06 | -0.32 | 0.38 | 0.25 |
| Bath | StPetes | 0.06 | -0.3 | 0.36 | 0.26 |
| Bath | Madras | 0.06 | 0.39 | 0.33 | 0.23 |
| StPetes | Madras | -0.3 | 0.39 | 0.69 | 0.25 |
| Harare | Madras | -0.32 | 0.39 | 0.71 | 0.24 |
| Harare | StPetes | -0.32 | -0.3 | 0.02 | 0.26 |

DIF is evident when the absolute difference is greater than the Dmin criterion.

Differential item functioning plots and post-doc Tukey test results for Item F20.2


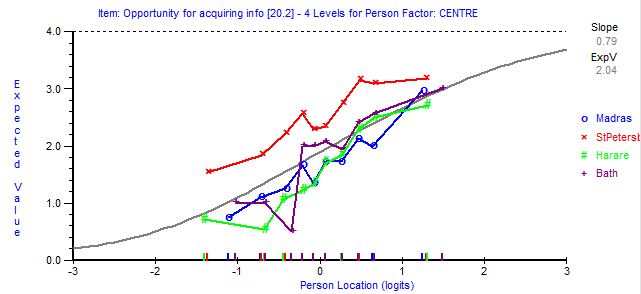


This figure shows the expected response to the item (*y-*axis) at each level across the continuum of quality of life (*x*-axis). Each country is plotted separately.

| Country 1 | Country 2 | Mean 1 | Mean 2 | Absolute Difference | Dmin criterion |
| --- | --- | --- | --- | --- | --- |
| Bath | Harare | 0.04 | -0.35 | 0.39 | 0.24 |
| Bath | StPetes | 0.04 | 0.7 | 0.66 | 0.25 |
| Bath | Madras | 0.04 | -0.25 | 0.29 | 0.22 |
| StPetes | Madras | 0.7 | -0.25 | 0.95 | 0.23 |
| Harare | Madras | -0.35 | -0.25 | 0.1 | 0.23 |
| Harare | StPetes | -0.35 | 0.7 | 1.05 | 0.25 |

DIF is evident when the absolute difference is greater than the Dmin criterion.

Differential item functioning plots and post-doc Tukey test results for Item F20.4


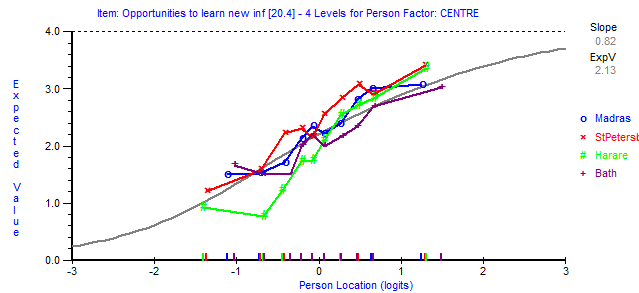


This figure shows the expected response to the item (*y-*axis) at each level across the continuum of quality of life (*x*-axis). Each country is plotted separately.

| Country 1 | Country 2 | Mean 1 | Mean 2 | Absolute Difference | Dmin criterion |
| --- | --- | --- | --- | --- | --- |
| Bath | Harare | -0.14 | -0.2 | 0.06 | 0.26 |
| Bath | StPetes | -0.14 | 0.29 | 0.43 | 0.27 |
| Bath | Madras | -0.14 | 0.08 | 0.22 | 0.24 |
| StPetes | Madras | 0.29 | 0.08 | 0.21 | 0.25 |
| Harare | Madras | -0.2 | 0.08 | 0.28 | 0.25 |
| Harare | StPetes | -0.2 | 0.29 | 0.49 | 0.27 |

DIF is evident when the absolute difference is greater than the Dmin criterion.

Differential item functioning plots and post-doc Tukey test results for Item F21.2


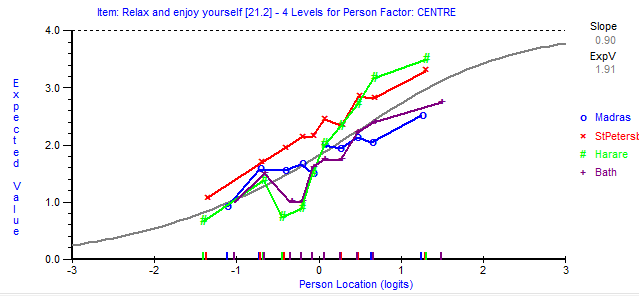


This figure shows the expected response to the item (*y-*axis) at each level across the continuum of quality of life (*x*-axis). Each country is plotted separately.

| Country 1 | Country 2 | Mean 1 | Mean 2 | Absolute Difference | Dmin criterion |
| --- | --- | --- | --- | --- | --- |
| Bath | Harare | -0.26 | -0.12 | 0.14 | 0.27 |
| Bath | StPetes | -0.26 | 0.47 | 0.73 | 0.28 |
| Bath | Madras | -0.26 | -0.09 | 0.17 | 0.26 |
| StPetes | Madras | 0.47 | -0.09 | 0.56 | 0.27 |
| Harare | Madras | -0.12 | -0.09 | 0.03 | 0.26 |
| Harare | StPetes | -0.12 | 0.47 | 0.59 | 0.29 |

DIF is evident when the absolute difference is greater than the Dmin criterion.
