# Supplementary material for: Adjusting for cross-cultural differences in computer-adaptive tests of quality of life
Source: Qual Life Res. 2017 Dec 4;27(4):1027–39. doi: 10.1007/s11136-017-1738-7 (PMC5874271; doi:10.1007/s11136-017-1738-7)
Supplement: Supplementary file 2 — Supplementary material 2 (DOCX 22 KB) [file 11136_2017_1738_MOESM2_ESM.docx]

**Appendix Two – Threshold values for all item banks**

Threshold values are relevant to this manuscript and may change over time, especially if additional countries are added to the item bank. Please contact the lead author at [drcgibbons@gmail.com](mailto:drcgibbons@gmail.com) for the latest parameter values if you plan to implement these item banks as CATs.
